# Supplementary material for: Enhanced Hydrogen Evolution Reactivity of T’-Phase Tungsten Dichalcogenides (WS2, WSe2, and WTe2) Materials: A DFT Study
Source: Int J Mol Sci. 2022 Oct 3;23(19):11727. doi: 10.3390/ijms231911727 (PMC9569713; doi:10.3390/ijms231911727)
Supplement: Supplementary file 1 [file ijms-23-11727-s001.zip › ijms-1948273-supplementary.pdf]

Supporting Information

Enhanced hydrogen evolution reaction activity of T′-phase tungsten dichalcogenides (WS_2, WSe_2, and WTe_2) materials by defect engineering

Haihua Huang [1], Guowei Hu [1], Chengchao Hu [1] and Xiaofeng Fan [2,*]

[1] School of Materials Science and Engineering, Liaocheng University, Liaocheng 252059, China
[2] College of Materials Science and Engineering, Jilin University, Changchun 130012, China
* Correspondence: xffan@jlu.edu.cn

Table S1. Hydrogen adsorption energy (E_{ads}) of T'-WS_2, T'-WSe_2 and T'-WTe_2 with stable various defect configurations at different adsorption positions.

	Defect	Sites	E_{ads} (eV)		Defect	Sites	Eads (eV)		Defect	Sites	Eads (eV)
WS_2	V1	-	-	WSe_2	V1	-	-	WTe_2	V1	-	-
		-	-			-	-			-	-
	V2	top-V	-3.06		V2	top-V	-3.10		V2	top-V	-2.95
		top-S1	-2.16			top-Se	-1.77			top-Te	-1.55
	DV1	-	-		DV1	-	-		DV1	-	-
		-	-			-	-			-	-
	DV2	top-V	-2.96		DV2	top-V	-3.01		DV2	top-v1	-2.91
		top-S1	-1.71			top-Se	-1.65			top-v2	-2.51
	B_S	top-B	-3.09		B_{Se}	top-B	-3.13		B_{Te}	top-B	-3.04
		top-S1	-2.33			top-Se	-1.84			top-Te	-1.62
	C_S	top-C	-3.14		C_{Se}	top-C	-3.24		C_{Te}	top-C	-3.23
		top-S2	-2.24			top-Se	-1.79			top-Te	-1.68
	P_S	top-P	-2.89		P_{Se}	top-P	-2.78		P_{Te}	top-P	-2.69
		top-S2	-2.20			top-Se	-1.83			top-Te	-1.68

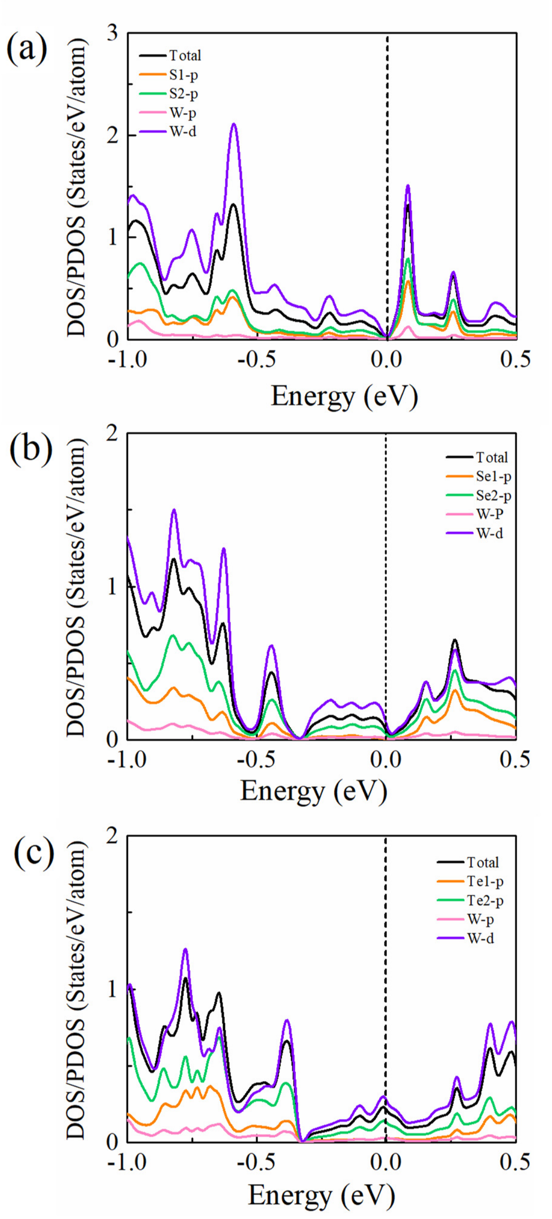

Figure S1. Electronic density of states of (a) T'-WS_2, (b) T'-WSe_2 and (c) T'-WTe_2, respectively. The vertical dot line denotes the fermi level.

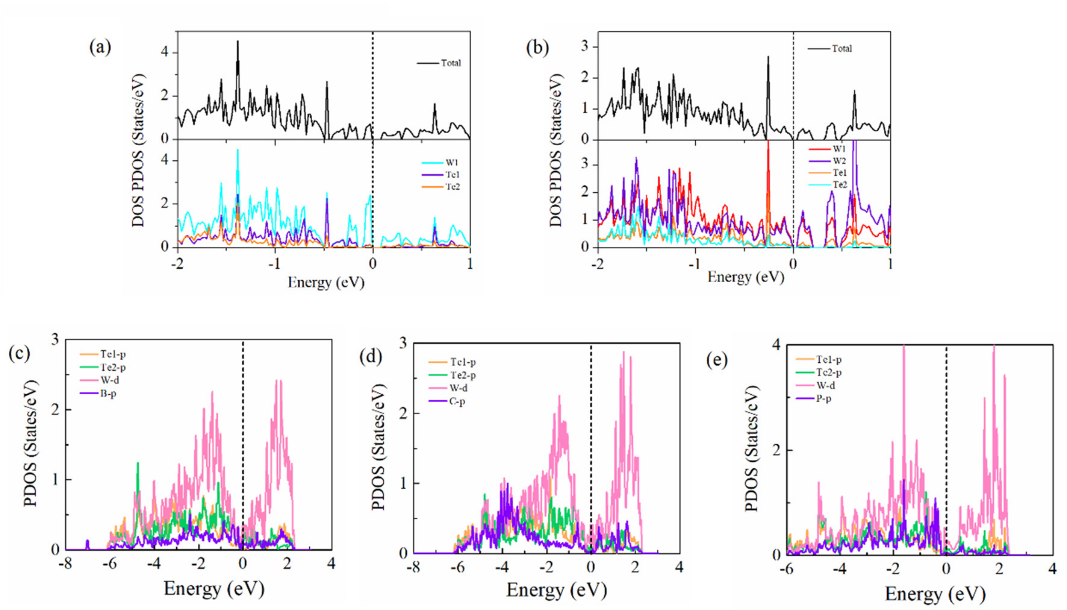

Figure S2. Density of states (DOS) and partial density of states (PDOS) of T'-WTe_2 monolayer with (a) single-vacancy (V), (b) double-vacancy (DV), (c) B-doping, (d) C-doping and (e) P-doping, respectively. The vertical dotted line denotes the fermi level.

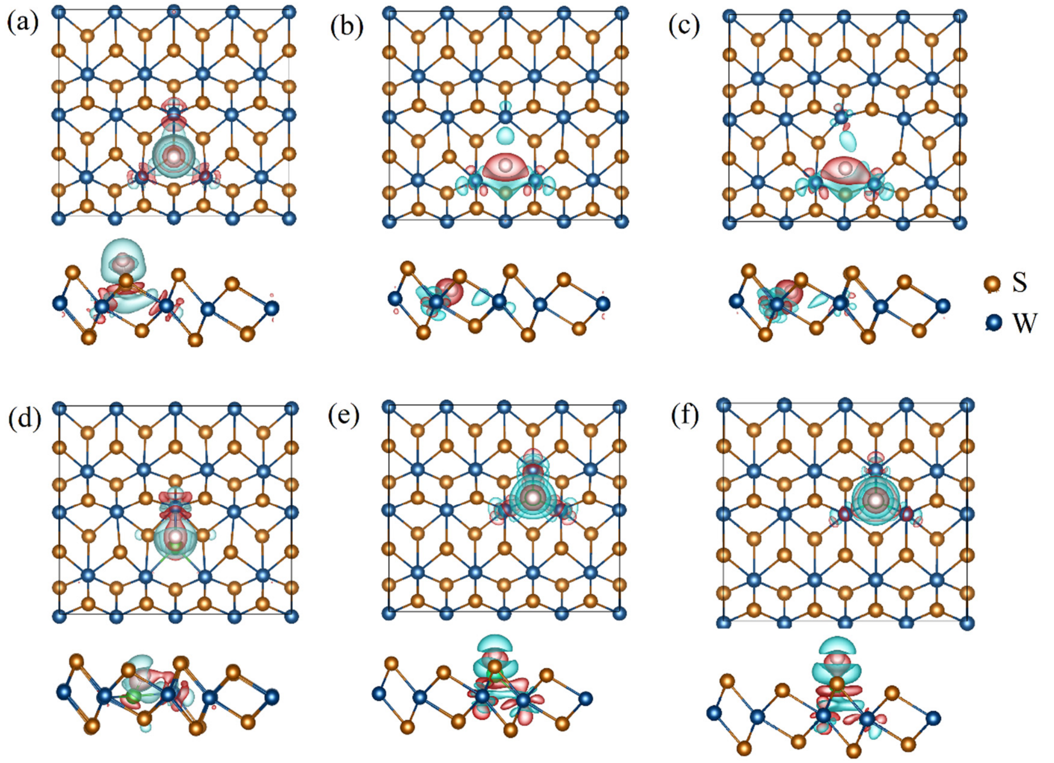

Figure S3. Difference of charge density of WS_2 with H-adsorption system for (a) pristine, (b) single-vacancy, (c) double-vacancy, (d) B-doping, (e) C-doping and (f) P-doping from top and side view, respectively. Note that red color indicates charge accumulation and green color denotes charge depletion.

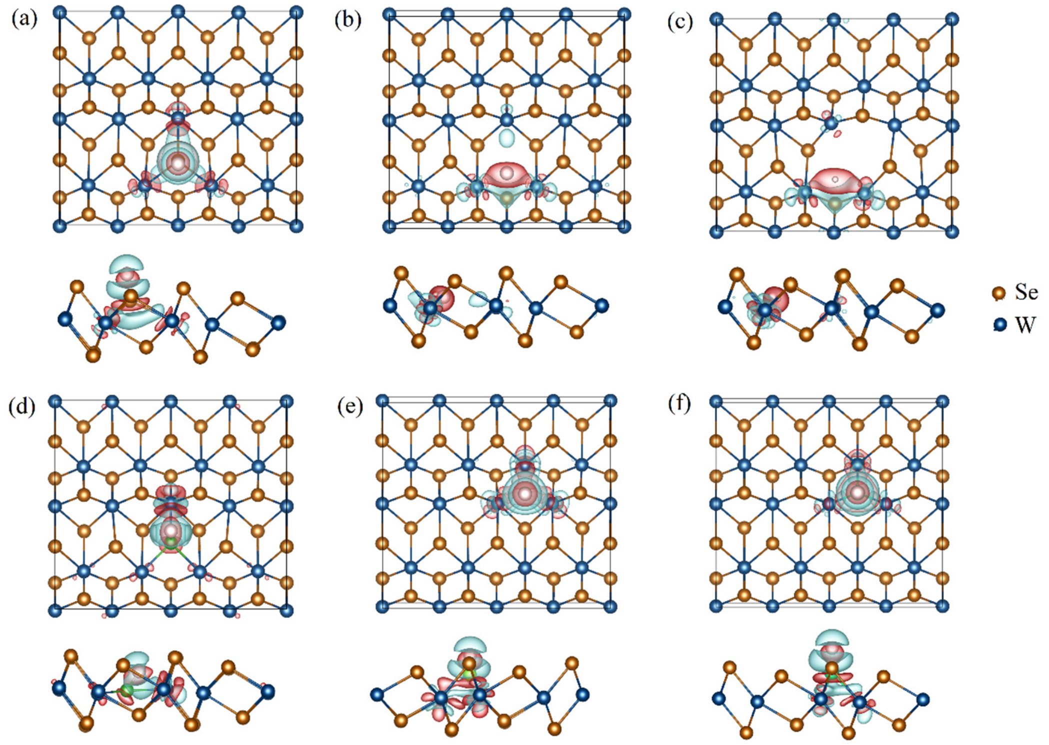

Figure S4. Difference of charge density of WSe_2 with H-adsorption system for (a) pristine, (b) single-vancancy, (c) double-vacancy, (d) B-doping, (e) C-doping and (f) P-doping from top and side view, respectively. Note that red color indicates charge accumulation and green color denotes charge depletion.
